# Evaluating the Stability of RNA-Seq Transcriptome Profiles and Drug-Induced Immune-Related Expression Changes in Whole Blood

**DOI:** 10.1371/journal.pone.0133315

**Published:** 2015-07-15

**Authors:** John F. Bowyer, Karen M. Tranter, Joseph P. Hanig, Nathaniel M. Crabtree, Robert P. Schleimer, Nysia I. George

**Affiliations:** 1 Division of Neurotoxicology, National Center for Toxicological Research, United States Food and Drug Administration, Jefferson, Arkansas, United States of America; 2 Division of Bioinformatics and Biostatistics, National Center for Toxicological Research, United States Food and Drug Administration, Jefferson, Arkansas, United States of America; 3 Center for Drug Evaluation and Research, United States Food and Drug Administration, Silver Spring, Maryland, United States of America; 4 Division of Allergy and Immunology, Northwestern Feinberg School of Medicine, Chicago, Illinois, United States of America; AC Camargo Cancer Hospital, BRAZIL

## Abstract

Methods were developed to evaluate the stability of rat whole blood expression obtained from RNA sequencing (RNA-seq) and assess changes in whole blood transcriptome profiles in experiments replicated over time. Expression was measured in globin-depleted RNA extracted from the whole blood of Sprague-Dawley rats, given either saline (control) or neurotoxic doses of amphetamine (AMPH). The experiment was repeated four times (paired control and AMPH groups) over a 2-year span. The transcriptome of the control and AMPH-treated groups was evaluated on: 1) transcript levels for ribosomal protein subunits; 2) relative expression of immune-related genes; 3) stability of the control transcriptome over 2 years; and 4) stability of the effects of AMPH on immune-related genes over 2 years. All, except one, of the 70 genes that encode the 80s ribosome had levels that ranked in the top 5% of all mean expression levels. Deviations in sequencing performance led to significant changes in the ribosomal transcripts. The overall expression profile of immune-related genes and genes specific to monocytes, T-cells or B-cells were well represented and consistent within treatment groups. There were no differences between the levels of ribosomal transcripts in time-matched control and AMPH groups but significant differences in the expression of immune-related genes between control and AMPH groups. AMPH significantly increased expression of some genes related to monocytes but down-regulated those specific to T-cells. These changes were partially due to changes in the two types of leukocytes present in blood, which indicate an activation of the innate immune system by AMPH. Thus, the stability of RNA-seq whole blood transcriptome can be verified by assessing ribosomal protein subunits and immune-related gene expression. Such stability enables the pooling of samples from replicate experiments to carry out differential expression analysis with acceptable power.

## Introduction

From a clinical standpoint, transcriptomic profiling of human blood (peripheral), or its various cells types, can be a useful tool for identifying biomarkers related to disease, immune response, and exposure to irritants [1–5]. Transcriptome analysis of blood in animal studies has also shown potential for identifying candidate blood-based biomarkers [6]. Until recently, much of the prior research has been conducted using microarray technologies. However, due to greater precision, increased coverage, and larger dynamic range, RNA sequencing (RNA-seq) platforms offer a competitive alternative to analyze changes in transcriptome expression [7]. Although whole blood analysis faces a unique set of challenges because globin mRNA depletion is necessary to ensure data quality, the purported advantages of RNA-seq technologies in terms of dynamic range and novel differential expression have also been observed in whole blood transcriptome studies [2,8]. These researchers found that despite some of the advantages of RNA-seq over microarrays, RNA sequencing reported greater within-group variability in whole blood. Typically, RNA-seq expression demonstrates higher reproducibility and lower variability in genes with high expression [7,9,10] but globin reduction can affect signal variability in whole blood. Thus, we evaluated the stability of globin-depleted mRNA from whole blood RNA-seq expression over time. Since RNA-seq technologies have been approved for clinical use by the FDA [11], changes in blood transcriptome due to toxicity in laboratory animals will play a large role in translating such changes to human data.

Globin transcripts compose over 70% of the expressed mRNA signal isolated from whole blood, and their removal has been reported to increase the integrity of transcript expression [12,13]. However, it has also been suggested that globin transcript depletion affects RNA yields, which would likely affect expression profiles [13]. As an additional means of assessing the overall stability of globin-depleted whole blood RNA-seq profiles, we have studied two sets of important comparisons. One set of comparisons is based on subunits of the 80s ribosome. In humans and mammals, the 80s ribosome consists of over 70 protein subunits, each of which is encoded by its own specific transcript [14]. These subunits are relatively unaffected by most drug treatments and experimental manipulations and are often used as “housekeeping” genes for RT-PCR [15–18]. Because all the subunits are necessary for 80s ribosomal function, their transcripts should be expressed at high levels (relative to the entire transcriptome).

The second comparison profiles transcripts found in the various types of leukocytes (white blood cells). Here, we compared the expression of transcripts specific for monocytes/macrophages, lymphocytes (T-cells and B-cells), and all leukocytes. In rat blood, there are roughly 10-times as many B- and T-cells (approximately equal in number) as monocytes. B-cells and T-cells make up approximately 80% of all leukocytes in rat blood and monocytes comprise only about 3%. Thus, one might suppose that the expression of monocyte specific transcripts to those specific for T-cells or B-cells might be 10-fold less and 30-fold less than transcripts expressed in all leukocytes. However, such an assumption is not correct for the chemokine, C-C motif, receptor 1 (*Ccr1*) since this gene is expressed at very high levels in monocytes. In contrast to this, neutrophils consist of over 10 to 20% of the white blood cells (WBC) in the circulating blood of rat but have relatively low levels of mRNA present, as is the case with most end stage cells including eosinophils and basophils [19].

The second primary objective of this study was to assess the stability of control and drug-treated transcriptome expression in the whole blood of Sprague-Dawley rats over time. Many laboratories often replicate the same experiment over time to ensure reproducibility of significant changes in expression resulting from drug or toxicant exposure. Generally, data from all time points are then pooled. However, when detecting modest changes (e.g. 1.5- to 3-fold), pooling data can be problematic if the measured endpoints fluctuate by 2-fold or more over time in the control group. In the current study, stability was assessed across four time points (ranging over a 2 year period) for a control group and a treatment group. Stability was evaluated primarily through comparisons of the expression profile of ribosomal subunit proteins and secondarily through comparisons profiling the entire transcriptome.

## Methods

### Animal Dosing and Sacrifice

This study was carried out in accordance with the declaration of Helsinki and the Guide for the Care and Use of Laboratory Animals as adopted and promulgated by the National Institutes of Health. The use of animal testing in this study was done under protocols E7295 and E7519 (issued to John Bowyer) that were approved by the NCTR institutional animal care and use committee (IACUC) which is fully accredited (Food and Drug Administration—National Center for Toxicological Research Accreditation #A4310-01) by NIH-OLAW. Nine- to ten-week-old (≈ 65 days) male Sprague-Dawley rats, ninety five total, were obtained from the Charles River Laboratories [Crl:CD(SD)]. Upon arrival at NCTR they received tail tattoos for identification. Prior to testing, rats were housed 2 per cage with food and water available ad libitum. Rats were housed on a daily 12 hr light cycle with lights on at 6:00 am and off at 6:00 pm. During housing, the temperature (23°C) and humidity (53%) were controlled. The rats were tested between 12 and 13 weeks of age (≈ 87 days old).

The data used in this study (a cohort of a larger study contained in GSE62368 and GSE64778) is comprised of data from *n* = 22 saline dosed “control” rats and *n* = 21 rats given a neurotoxic exposure to AMPH. The larger study was carried out to identify mRNA biomarkers and to determine transcriptional immune-related responses present in circulating blood after neurotoxic exposures to hyperthermia, neurotoxic AMPH exposure, and non-neurotoxic AMPH exposure. Herein, we focus solely on the control and neurotoxic AMPH treatment groups. Multiple replicates of the same experiment were conducted over a 2 year period. This was done to ensure that any significant effects seen in transcript expression would be robust and reproducible over time and hopefully subsequently observable in other laboratories. The sample size of the control group at each time period was: Con1: *n* = 3, Con2: *n* = 7, Con3: *n* = 6, and Con4: *n* = 6. The size of the AMPH groups were: AMPH1: *n* = 5, AMPH2: *n* = 3, AMPH3: *n* = 6, and AMPH4: *n* = 7. The saline control and AMPH groups were sacrificed on: Con1 and AMPH1, September 2012; Con2 and AMPH2, June 2013; Con3 and AMPH3, Aug 2013; Con4 and AMPH4, July 2014.

Dosing commenced at 8:00 a.m. and ended at 2:00 p.m. AMPH-exposed animals were given 4 doses of amphetamine comprised of a sequential exposure to 5, 7.5, 10, and 10 mg/kg AMPH subcutaneously with 2 hr between each dose. The d-amphetamine-sulfate (Sigma-Aldrich, St. Louis, MO) dose was dissolved in normal saline (1 ml/ kg injected). This AMPH dosing paradigm was modified slightly from an original acute methamphetamine dosing paradigm that was shown to produce neurotoxicity [20,21]. The saline animals received 4 injections of 1 ml/kg s.c. In all groups, the behavior and body temperature of each rat were monitored hourly during saline or AMPH exposure and until at least 3 hr after the last dose (time of sacrifice). The lethal effects of hyperthermia and hyperpyrexia in the AMPH groups when body temperatures exceeded 41.4°C were prevented by placing the animals unrestrained on crushed ice for 15 to 30 min in a clean, wood chip free cage to allow their temperatures to drop below 40.0°C.

### Collection and processing of cardiac blood

All rats were sacrificed at 4 to 5 pm with an overdose of 300 mg/kg body weight of pentobarbital, resulting in deep anesthesia in less than 3 min. At that point, 3 to 5 ml of cardiac blood was withdrawn from the heart using an 18 gauge needle attached to a 5 ml syringe, and the rats were then euthanatized with decapitation. Approximately 1 ml of the blood was immediately separated into approximately three 300 μl aliquots, frozen on dry ice in cylindrical foil capsules, and then stored at -70°C for later RNA-seq analysis. The remainder of the blood was injected into a 7.0 ml BD (Franklin Lanes, NJ) Vacutainer containing 12 mg EDTA (data from these aliquots are to be reported at later date). One ml was used to count the total RBC and WBC numbers as well as the different types of WBC present. Within 12 h of collection of the cardiac blood in EDTA coated vacutainers, one ml was used to determine total RBC, WBC counts, and the numbers of lymphocytes (T- and B-cells), monocytes, neutrophils, basophils and eosinophils present. Complete blood counts were determined on an ABX Pentra 60 C+ analyzer (ABX, Irvine CA). Maintenance and calibration was done according to the manufacturer’s recommendations. Three levels of assayed controls were included in daily analyses as internal controls.

### RNA isolation and RNA-seq processing

RNA isolation was performed using RNAzol BD (Molecular Research Center, Inc., www.mrcgene.com) [9] with modified procedures for frozen blood. Just prior to RNA isolation, the frozen 300–400 μl RBC or whole blood aliquots were placed on dry ice and cut into approximately 100 to 150 μl sections. Two or three of these smaller sections were immediately (were not allowed to thaw) homogenized in 1.6 ml of RNAzol BD containing 45μl of 5 N acetic acid using a 2 ml Teflon pestle homogenizer. Approximately 300 to 400 μl of frozen whole blood was used for RNA isolation. Separation of the upper phase containing the extracted RNA with lower phases containing protein and DNA was accomplished by adding 350 μl of 4-bromoanisole and centrifugations at 12,000 x g for 10 min. To precipitate the RNA, 600 μl 2-propanol was added to 600 to 700 μl the extracted upper phase. After 15 min, centrifugation at 12,000 x g was performed. The RNA ppt was washed once with 70% ethanol and water and a second wash of 100% ethanol. After ≈ 1 min air drying the RNA was dissolved in 25 to 35 μl of RNase-free water. The final total cellular RNA recovered ranged from 5 to 25 μg and was stored at -70°C until shipment for data processing. Two to four μl of each aliquot was set aside for purity analysis at NCTR using an Agilent 2200 TapeStation System (Agilent Technologies, Palo Alto, CA).

The isolated total RNA was then shipped overnight on dry ice to Expression Analysis Inc. [EA; Durham, NC] for globin transcript removal and sequencing. Alpha and Beta Globin mRNA were substantially depleted from total RNA samples using the GlobinClear-Mouse/Rat Kit (Life Technologies # AM1981), essentially as described by the vendor. Briefly, 1.25 μg of total RNA isolated from whole blood was combined with biotinylated capture oligonucleotides complementary to globin mRNAs and the mixture incubated at 50°C for 15 minutes to allow duplex formation. Streptavidin magnetic beads were added to each specimen, and the resulting mixture was incubated for an additional 30 minutes at 50°C to allow binding of the biotin moieties by Streptavidin. These complexes, comprising Streptavidin magnetic beads bound to biotinylated oligonucleotides that are specifically hybridized to globin mRNAs, were then captured using a magnet. The globin-depleted supernatant is transferred to a new container and further purified using RNA binding beads. The final globin mRNA-depleted RNA samples are quantitated by spectrophotometry using a NanoDrop ND-8000 spectrophotometer.

### RNA-seq Expression Profiling

EA created the library and performed 50bp paired-end and strand-specific sequencing using an Illumina platform. A total of 25–35 million reads were generated per sample. Sequencing by synthesis methods, as implemented via Illumina technology, were used to generate the RNA-seq data. Globin mRNA-depleted RNA samples were converted into cDNA libraries using the TruSeq Stranded mRNA Sample Prep Kit (Illumina, #RS-122-2103). Starting with 300 ng of globin mRNA-depleted RNA, polyadenylated RNA (primarily mRNA) was selected and purified using oligo-dT conjugated magnetic beads. This mRNA was chemically fragmented and converted into single-stranded cDNA using reverse transcriptase and random hexamer primers, with the addition of Actinomycin D to suppress DNA-dependent synthesis of the second strand. Double-stranded cDNA was created by removing the RNA template and synthesizing the second strand in the presence of dUTP in place of dTTP. A single A base was added to the 3’ end to facilitate ligation of sequencing adapters, which contain a single T base overhang. Adapter-ligated cDNA was amplified by polymerase chain reaction to increase the amount of sequence-ready library. During this amplification the polymerase stalls when it encounters a U base, rendering the second strand a poor template. Accordingly, amplified material used the first strand as a template, thereby preserving the strand information. Final cDNA libraries were analyzed for size distribution using an Agilent 2200 TapeStation (D1000 Screentape, Agilent # 5067–5582), quantitated by qPCR (KAPA Library Quant Kit, KAPA Biosystems # KK4824), then normalized to 2 nM in preparation for sequencing.

The 2nM normalized samples were pooled. cDNA templates were denatured using fresh 0.1N NaOH, and then diluted to a final loading concentration of 13pM. Cluster generation was performed on an Illumina cBOT (v1.5.12.0) using an Illumina TruSeq Paired-End Cluster Kit v3 (Illumina # PE-401-3001) to cluster an Illumina Paired End Flow Cell. Templates were attached to the flowcell via a dense lawn of oligonucleotides that bind to the sequencing adapters added during sample preparation, which are extended and then denatured. The flowcell was then sequenced through 51 bases, paired end, with an 8 base index cycle on an Illumina HiSeq 2000 (HiSeq Control Software v 1.5.15.1). During sequencing cycles, fluorescent reversible terminator dNTPs were added to the clusters, with only a single base per target being incorporated. Following imaging of the clusters, the terminator and fluorescent tag were cleaved so that the next base could be incorporated. Quality control files contained read length and depth results (before and after clipping), presence of artifact/ duplicate sequences, distribution of base quality and base frequency by sample. Also, flow cell total yield, PF reads, barcode quality, alignment summaries and number of genes detected were determined. The basecall files were converted to fastq files using CASAVA 1.8.2. The fastq files were clipped using fastq-mcf with the parameters “—max-ns 4—qual-mean 25-H-p 5-q 7-l 25” [22]. The fastq files were aligned to the rat Ensembl release 70 transcriptome (rn5) using bowtie 0.12.9 with the parameters “-e 500 –m 100 –chunkmbs 256”. The alignments were quantified using RSEM v 1.2.0 with no special parameters.

### RNA-seq Data Analysis

RNA-seq by Expectation-Maximization (RSEM) [23] data generated by EA was rounded to produce digital expression values. In total, 16,881 transcripts were mapped to the reference rat genome in all samples. Transcript-level outliers within each experimental group were identified by the iLOO method [24] and subsequently replaced with the trimmed mean. All reports of average transcript expression are summarized using DESeq2 normalized counts [25]. DESeq2 was also used to carry out differential expression analysis. Significance was assessed using p-values adjusted by the Benjamini-Hochberg method for multiple comparison testing. Transcripts with adjusted p-values lower than 0.05 and absolute log_2_(FC) values exceeding specific cutoffs were declared significant. All reported values of correlation reflect Pearson’s correlation coefficient and are significant at α = 0.05.

A number of criteria were used to identify the most stable transcripts for RNA-seq data. Ideally, a stable transcript would show minimal variability, be consistently expressed in the high region of expression, and demonstrate consistent expression across treatment groups. Thus, candidates for stable expression were transcripts with no detected outliers, no measurements in the low expressed region [26], and no significant difference between the control and treatment group.

All analyses were carried out in R (http://www.r-project.org).

## Results

The RNA integrity number (RIN) for the control whole blood samples (n = 22) ranged from 7.0 to 9.4, averaging 8.3 ± 0.15 (mean ± SEM). The RIN for the AMPH group ranged from 6.9 to 9.3, averaging 8.1 ± 0.15 (mean ± SEM), and did not significantly differ from control. Although globin depletion is reported to reduce expression [2], hemoglobin alpha (*Hba*) remained the highest expressed transcript. Also, as would be expected, the expression of olfactory receptor genes (approximately 1,200 unique transcripts [10]) was virtually absent in blood with only 14 genes having counts greater than 1. In addition, we assessed whether high levels of myoglobin transcript (*Mb*) were present in the blood samples. Since cardiac muscle contains very high levels of myoglobin [27], high expression of the myoglobin transcript could indicate that cardiac muscle was “contaminating” the samples as a result of the cardiac punctures to obtain whole blood. Two of the control animals from the Con4 group had very high levels (>5000 counts) for *Mb*. They were removed from further analysis resulting in n = 20 for the control group. None of the animals in the AMPH group had to be removed from the study due to high *Mb* levels. Mb expression for the remaining control and AMPH animals was relatively low with mean expressions of 29.8 ± 3.4 and 35.8 ± 1.8, respectively.

**Fig 1:**
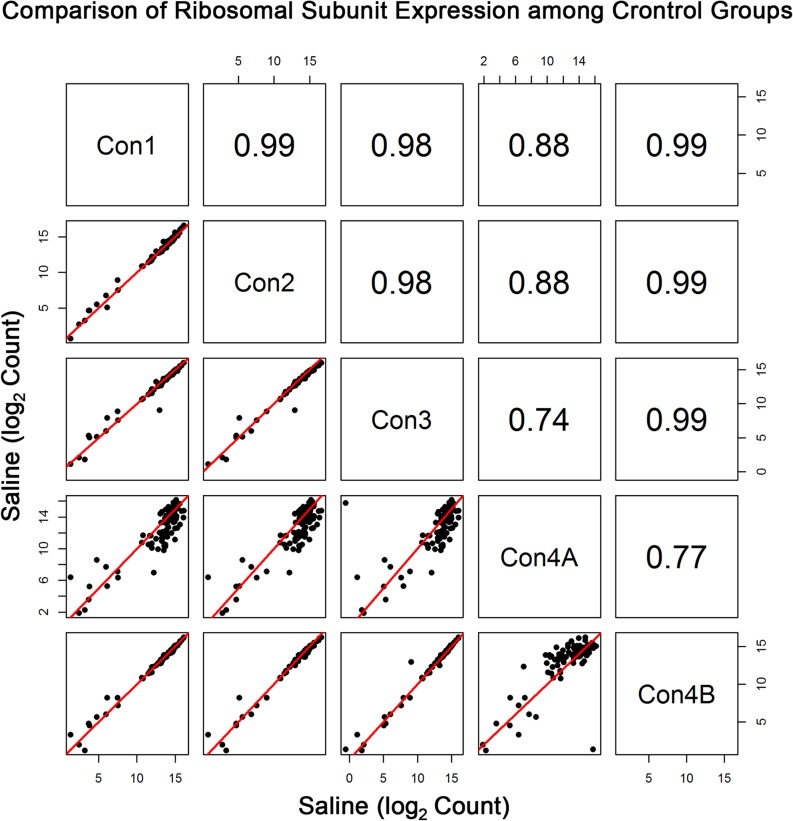
Comparison of the Ribosomal Subunit Expression among Control Groups. Pearson correlation and scatterplot matrix of log2 normalized expression of the ribosomal subunit proteins in control groups, Con1-Con4B. The red solid line is the identity line. Note that the aberrant/anomalous results of Con4A are due to less than optimal amplification prior to sequencing.

Average expression for the 70+ genes necessary to encode the 80s ribosome are shown in Table 1. The large 70s subunit transcripts are labeled with an NCBI gene symbol beginning with “*Rpl*,” while the small 30s subunit transcripts begin with “*Rps*.” Average expression for most of the transcripts was ranked in the top 5^th^ percentile of expression levels (i.e. rank of 844 or higher). All of the ribosomal subunit genes, except Rpl22, had at least one transcript (splice variant) in the top 5^th^ percentile; Rpl22 ranked 1010 in whole blood. RNA-seq expression of the ribosomal proteins for control animals were highly correlated (r≥0.98 for log2 expression data) across the first 3 time points (Fig 1).

**Table 1:** Expression of Rat Ribosomal Subunit Transcripts in the Whole Blood of Control and Amphetamine Treated Rats.

	Saline Control			Amphetamine Treated			
			Expression			Expression	AMPH/
NCBI Gene Symbol	Mean ± S.E.M.		Rank	Mean ± S.E.M.		Rank	Saline Control
Rpl10:ENSRNOG00000027149	31893.0 ±	421.9	105	34635.3 ±	401.4	98	1.3
Rpl10a:ENSRNOG00000000505	9887.6 ±	100.5	334	8928.4 ±	127.1	369	1.2
Rpl11:ENSRNOG00000026260	23073.5 ±	289.6	150	24913.7 ±	391.7	154	1.4
Rpl12:ENSRNOG00000016220	15528.6 ±	365.5	224	15192.8 ±	289.8	248	1.4
*Rpl12:ENSRNOG00000028993	7674.5 ±	167.0	401	7969.2 ±	210.0	408	1.2
*Rpl12:ENSRNOG00000029115	15150.2 ±	247.8	228	14472.4 ±	220.1	256	1.3
Rpl13:ENSRNOG00000015335	29819.8 ±	415.3	117	31336.5 ±	491.6	109	1.5
Rpl13a:ENSRNOG00000020618	28861.6 ±	308.3	120	29432.5 ±	507.0	121	1.4
Rpl14:ENSRNOG00000019007	12413.0 ±	141.1	274	9284.6 ±	112.9	356	0.9
Rpl15:ENSRNOG00000008140	15195.2 ±	166.8	227	13822.1 ±	183.4	263	1.2
Rpl17:ENSRNOG00000018680	21339.7 ±	288.1	167	19123.8 ±	214.3	198	1.1
Rpl18:ENSRNOG00000021035	8567.6 ±	81.7	372	8457.1 ±	138.5	389	1.4
Rpl18a:ENSRNOG00000018795	40188.2 ±	592.3	76	51024.5 ±	1097.5	64	1.8
Rpl19:ENSRNOG00000004741	25006.9 ±	290.9	135	23030.5 ±	352.5	169	1.2
Rpl21:ENSRNOG00000032803	16950.7 ±	275.5	209	16430.0 ±	243.4	226	1.0
Rpl22:ENSRNOG00000011104	2727.7 ±	31.8	1010	2837.7 ±	48.3	983	1.4
Rpl23:ENSRNOG00000004107	13309.8 ±	134.4	259	11163.5 ±	164.0	311	0.9
Rpl23a:ENSRNOG00000023344	17242.6 ±	186.9	205	14001.2 ±	178.8	260	0.9
Rpl24:ENSRNOG00000001611	22081.8 ±	302.1	155	23210.1 ±	337.5	165	1.4
Rpl26:ENSRNOG00000004214	31889.1 ±	499.8	106	33986.3 ±	570.6	101	1.3
Rpl27:ENSRNOG00000020674	7331.6 ±	71.5	415	6345.6 ±	69.5	505	1.1
Rpl27a:ENSRNOG00000014214	24434.3 ±	302.1	140	25924.0 ±	476.5	146	1.3
Rpl29:ENSRNOG00000011138	14791.7 ±	189.7	234	14602.4 ±	199.2	255	1.4
Rpl3:ENSRNOG00000016896	14420.0 ±	141.7	241	8603.1 ±	138.8	385	0.7
Rpl30:ENSRNOG00000005975	13539.3 ±	167.3	255	13097.6 ±	206.3	274	0.9
Rpl31:ENSRNOG00000013508	24249.5 ±	349.2	142	23430.2 ±	332.7	163	1.2
Rpl32:ENSRNOG00000010746	28258.2 ±	396.6	122	28800.5 ±	432.8	128	1.4
Rpl34:ENSRNOG00000016387	8489.6 ±	87.5	375	7677.0 ±	113.7	425	1.2
*Rpl34:ENSRNOG00000038045	28.2 ±	0.7	10918	31.5 ±	0.9	10455	1.5
Rpl35:ENSRNOG00000014272	33448.1 ±	591.9	99	35533.8 ±	514.4	94	1.5
Rpl35a:ENSRNOG00000031641	17519.3 ±	225.7	200	17619.4 ±	239.9	214	1.2
Rpl36:ENSRNOG00000033473	4599.4 ±	54.8	608	5142.6 ±	135.7	581	1.6
Rpl36a:ENSRNOG00000011494	3296.0 ±	32.0	833	2592.8 ±	42.7	1055	1.0
*Rpl36a:ENSRNOG00000031315	3262.0 ±	35.3	846	2692.5 ±	50.0	1026	1.0
Rpl37:ENSRNOG00000012538	38285.2 ±	804.7	81	47356.1 ±	957.2	72	1.7
*Rpl37:ENSRNOG00000033803	6.3 ±	0.4	13020	6.1 ±	0.2	12923	1.2
Rpl37a:ENSRNOG00000023385	19229.3 ±	296.9	186	23055.8 ±	452.0	167	1.6
*Rpl37a:ENSRNOG00000028883	4612.0 ±	61.7	606	6254.9 ±	146.9	512	1.8
Rpl38:ENSRNOG00000036729	34474.2 ±	989.8	93	42146.7 ±	1131.0	81	1.5
Rpl39:ENSRNOG00000043348	9446.1 ±	130.5	343	10128.0 ±	165.6	334	1.0
*Rpl39:ENSRNOG00000050074	1729.5 ±	22.5	1556	1868.1 ±	32.6	1424	1.3
Rpl4:ENSRNOG00000009378	45765.1 ±	487.7	70	38909.3 ±	557.6	87	1.0
Rpl41:ENSRNOG00000042233	80725.2 ±	1496.6	43	109715.4 ±	2532.4	36	1.8
Rpl5:ENSRNOG00000023529	24050.0 ±	243.6	143	16129.6 ±	204.8	233	0.7
Rpl6:ENSRNOG00000025936	10268.5 ±	78.2	322	7202.2 ±	126.3	445	0.8
*Rpl6:ENSRNOG00000031889	3282.0 ±	36.0	838	1904.4 ±	31.8	1401	0.8
Rpl7:ENSRNOG00000006992	29977.4 ±	327.1	116	29814.7 ±	425.4	118	1.1
Rpl8:ENSRNOG00000032635	13255.6 ±	118.4	261	11086.7 ±	164.8	314	1.2
Rpl9:ENSRNOG00000026716	17304.3 ±	159.2	204	13393.7 ±	205.0	272	0.8
*Rpl9:ENSRNOG00000030476	153.6 ±	11.2	7710	264.4 ±	15.2	5606	2.7
Rplp0:ENSRNOG00000001148	73659.3 ±	1015.5	47	81630.6 ±	1118.8	44	1.5
Rplp1:ENSRNOG00000013874	40.9 ±	1.3	10316	79.4 ±	5.8	8646	0.9
*Rplp1:ENSRNOG00000011955	55821.4 ±	911.5	62	74504.6 ±	1882.8	49	1.9
Rplp2:ENSRNOG00000002116	17380.8 ±	256.1	203	19606.5 ±	394.2	194	1.6
Rps10:ENSRNOG00000000490	13917.0 ±	208.8	252	17008.0 ±	395.3	218	1.7
Rps11:ENSRNOG00000020595	26964.8 ±	283.4	126	21665.1 ±	248.7	176	1.0
Rps12:ENSRNOG00000016411	41375.2 ±	662.4	72	49498.5 ±	824.3	69	1.4
Rps13:ENSRNOG00000020502	12198.2 ±	126.3	278	10438.1 ±	143.2	328	1.0
Rps14:ENSRNOG00000018774	26173.5 ±	364.3	129	36716.1 ±	987.6	93	1.8
Rps15:ENSRNOG00000024603	22360.2 ±	351.5	152	25440.3 ±	462.5	149	1.4
Rps15a:ENSRNOG00000018320	12646.9 ±	138.8	272	12070.6	215.2	296	1.1
Rps16:ENSRNOG00000019578	29497.0 ±	348.7	118	32065.2	590.8	106	1.4
Rps17:ENSRNOG00000019106	25283.8 ±	343.9	133	25954.4	418.2	145	1.3
*Rps17:ENSRNOG00000045885	5.2 ±	0.2	13271	6.1	0.2	12921	1.5
Rps18:ENSRNOG00000028505	21474.0 ±	239.0	166	18142.0	259.0	210	1.2
*Rps18:ENSRNOG00000033152	179.6 ±	3.4	7301	204.3	8.9	6314	1.5
Rps19:ENSRNOG00000037897	8822.0 ±	106.2	363	8943.1	123.9	368	1.4
*Rps19:ENSRNOG00000048199	8822.0 ±	106.2	363	8943.1	123.9	368	1.4
Rps2:ENSRNOG00000014179	38904.8 ±	702.2	80	42806.2	800.0	78	1.5
Rps20:ENSRNOG00000008555	5637.0 ±	234.6	514	4117.5	199.2	708	1.1
*Rps20:ENSRNOG00000029627	7704.9 ±	125.6	399	11610.1 ±	382.7	304	2.1
Rps21:ENSRNOG00000006325	10307.7 ±	119.1	319	10628.3 ±	201.3	323	1.4
Rps23:ENSRNOG00000016580	24662.4 ±	393.2	139	25586.8 ±	427.8	148	1.3
Rps24:ENSRNOG00000010189	28135.8 ±	370.4	123	30522.1 ±	547.9	113	1.1
*Rps24:ENSRNOG00000031579	1800.8 ±	21.9	1497	2110.4 ±	48.2	1282	1.2
Rps25:ENSRNOG00000027503	21631.1 ±	276.9	162	22315.4 ±	341.5	173	1.1
Rps26:ENSRNOG00000005517	11789.5 ±	130.0	288	11758.4 ±	172.6	300	1.4
Rps27:ENSRNOG00000016961	17810.5 ±	178.1	197	15471.2 ±	277.0	247	1.0
Rps27a:ENSRNOG00000004426	36526.0 ±	507.4	85	42300.5 ±	764.1	80	1.2
Rps28:ENSRNOG00000049442	25.6 ±	0.8	11066	42.2 ±	1.8	9919	1.8
*Rps28:ENSRNOG00000042886	6562.1 ±	71.4	450	6716.2 ±	122.8	477	1.4
Rps29:ENSRNOG00000004196	62022.7 ±	1182.1	51	77274.0 ±	1721.9	48	1.7
*Rps29:ENSRNOG00000028939	398.2 ±	8.3	4979	542.5 ±	18.5	3683	1.9
*Rps29:ENSRNOG00000029443	79.9 ±	2.3	9166	94.3 ±	3.5	8251	1.1
Rps3:ENSRNOG00000017418	16506.2 ±	146.5	213	12966.6 ±	159.7	278	1.0
Rps3a:ENSRNOG00000011893	34804.9 ±	382.8	92	32152.9 ±	478.1	105	0.9
Rps4x:ENSRNOG00000029574	0.7 ±	0.1	15754	67.2 ±	8.5	8981	16.2
Rps4x:ENSRNOG00000003201	32176.8 ±	387.7	104	30607.2 ±	498.8	112	1.3
Rps5:ENSRNOG00000019453	12298.5 ±	132.2	277	12072.3 ±	193.8	295	1.4
Rps6:ENSRNOG00000007663	20594.8 ±	220.2	175	14431.5 ±	162.7	257	0.9
*Rps6:ENSRNOG00000049025	7738.2 ±	88.5	397	7193.3 ±	179.5	446	0.9
Rps7:ENSRNOG00000008551	23143.1 ±	257.8	149	18565.1 ±	248.2	204	1.0
*Rps7:ENSRNOG00000027694	3.6 ±	0.3	13751	3.5 ±	0.3	13723	0.8
Rps8:ENSRNOG00000018768	36417.7 ±	577.2	86	34246.2 ±	498.0	99	1.2
Rps9:ENSRNOG00000011355	11785.5 ±	175.2	289	14631.5 ±	208.2	254	1.8
*Rps9:ENSRNOG00000049813	3203.4 ±	32.0	861	4394.3 ±	72.6	672	1.7

* Indicates that more than one transcript was expressed for this particular ribosomal subunit gene.

**Fig 2:**
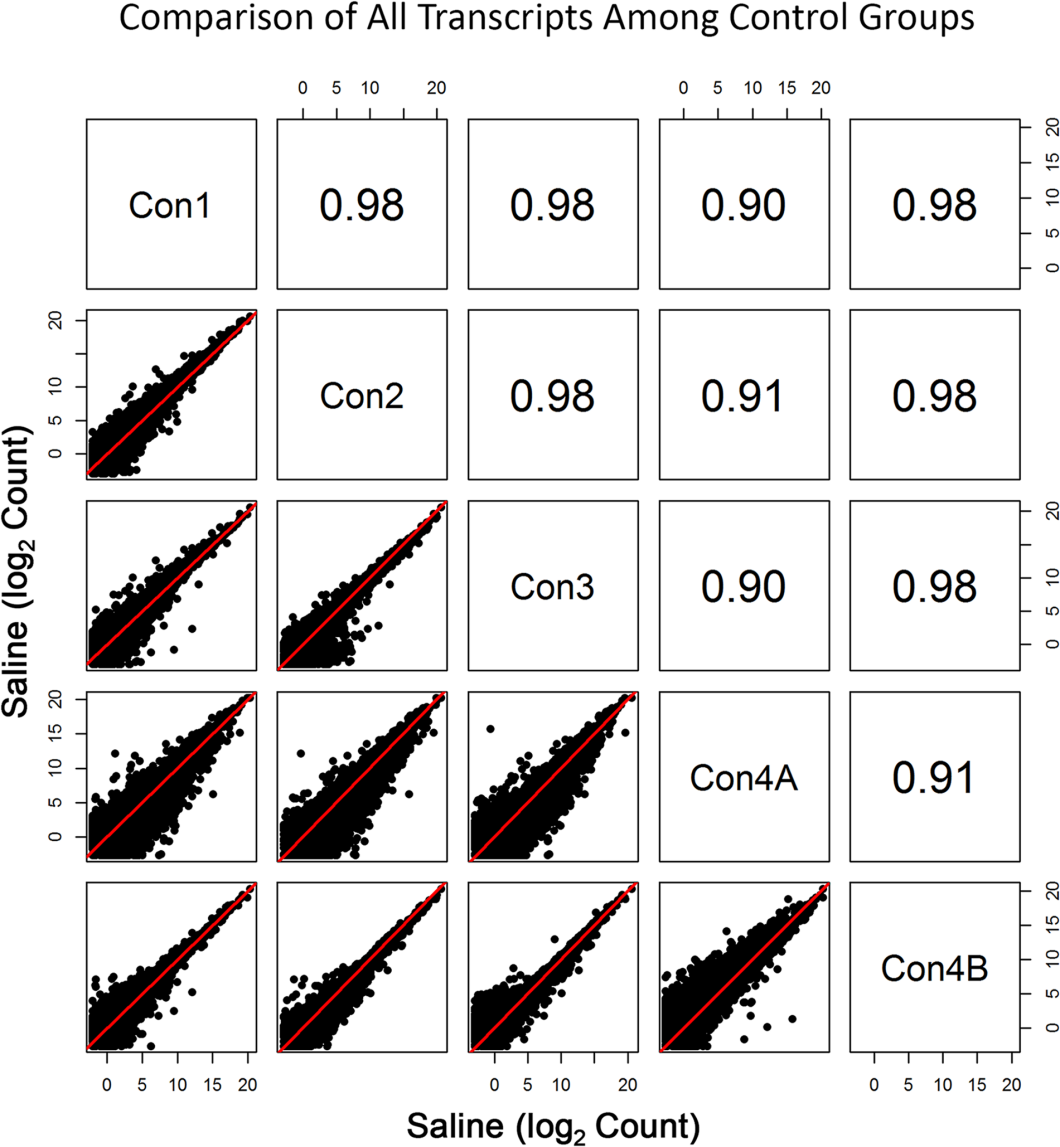
Comparison of All Transcripts among the Control Groups. Pearson correlation and scatterplot matrix of log2 normalized expression of all transcripts in control groups, Con1-Con4B. The red solid line is the identity line. Again, the aberrant results of Con4A are due to less than optimal amplification prior to sequencing.

Initial sequencing of the Con4 group (denoted by Con4A) yielded poorer correlation with the other groups (r≥0.74 for log2 expression data) (Fig 1). To some extent, diminished correlation between Con4A and Con1 – Con3 was also evident when assessing profiles of the entire transcriptome (Fig 2). Interestingly, this was the case despite the fact that total clusters, genes detected, % genes detected, % transcriptome mapped, skewness, median coverage, and % of all genes mapped did not differ between Con1, Con2, Con3, and Con4A. One noticeable fluctuation was the slight decrease in %(G+C), which ranged from 43% to 48% for Con4A, compared to the usual 48% to 53% range for the other groups. The less than optimal sequencing results shown with Con4A were due to an Illumina library preparation kit that contained a less than optimal PCR Master Mix reagent.

**Fig 3:**
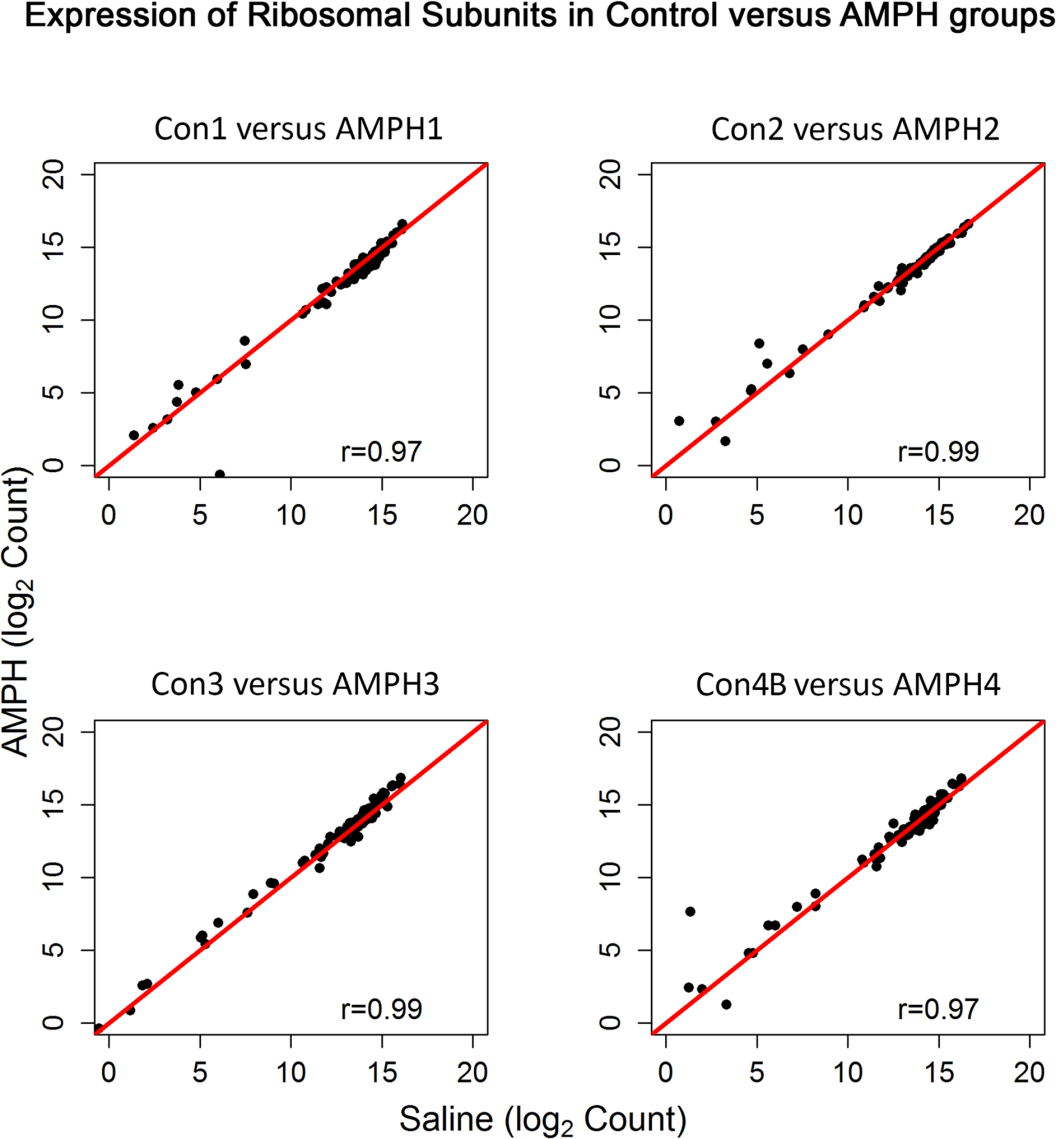
Expression of Ribosomal Subunits in the Control versus Amphetamine Groups. Pearson correlation and scatterplot matrix of log2 normalized expression of the ribosomal subunit proteins comparing each control group to its time-matched AMPH group. The red solid line is the identity line.

Subsequently, aliquots of the same RNA from the 4 animals in the Con4A group were re-sequenced (denoted by Con4B), yielding a more accurate expression of the ribosomal proteins (Fig 1) and comparable %(G+C) values. Differential expression (DE) analysis of pairwise comparisons of the four control groups indicated that error rates (i.e. DE transcripts) were well below the nominal level. For example, DE analysis comparing Con1 and Con4B, the most distant pairwise time points, returned only 3 significant transcripts. Thus, although the groups were dosed and sacrificed two years apart, stability of transcript expression in control animals was maintained. When profiles of the ribosomal subunit expression for the four AMPH groups (AMPH1, AMPH2, AMPH3 and AMPH4) were compared to each other, pairwise correlation values were comparable to those observed for the control groups (correlation values among the four pairs ranged between 0.97 and 0.98). Somewhat surprisingly, correlation between each control group and the time-matched AMPH group was at least 0.97 (Fig 3).

To further characterize repeatability of the control expression profiles, 29 genes specifically related to the immune system and found predominately on leukocytes were evaluated. The mean (and standard error) of each gene is reported in Tables 2 and 3 for both groups (all four subgroups were included in the calculations). Most of the selected genes are classified as cluster of differentiation antigens (i.e. classification determinant) and are located on the outer membranes of leukocytes. The seven transcripts primarily expressed on monocytes/macrophages (*Ccr1*, *Cd14*, *Cd163*, *Cd68*, *Itgax*, *Nos2* and *Arg1*) had a 1000-fold range if indeed interleukin 1 β is exclusively produced in monocytes (*Il1b* counts reached 18,000). Ten genes preferentially expressed in either T-cells (*Cd2*, *Cd3e*, *Cd8a*, *Cd28*, *Cd247*) or B-cells (*Cd19*, *Cd22*, *Cd79b*, *Cd180* and *Ly86*) had a median expression of 1921 with only slightly more than a 10-fold range. The median expression of 6 selected transcripts found in almost all leukocytes (*Cd300a*, *Cd44*, *Cd48*, *Cd84*, *Cd97* and *Sell*) was 6342 but ranged over 70-fold. The neutrophil related genes (*Camp*, *Elane*, *Mpo*, *Mme*, *Prtn3 and Ctsg*) had expression levels below 100 except for *Camp*.

**Table 2:** Expression of Selected Immune-Related Transcripts in Control and Amphetamine Treated Rats.

		Saline Controls		Amphetamine Treated		
	NCBI Gene Symbol	Mean ± S.E.M.	Expression Rank	Mean ± S.E.M.	Expression Rank	DE P-value
Primarily	*Ccr1*	6059.4 ± 282.2	484	7714.5 ± 131.9	424	N.S.
Expressed	*Itgax (Cd11c)*	1874.2 ± 24.6	1440	1976.7 ± 42.4	1358	0.0254
in Monocytes	*Cd14*	340.4 ± 12.4	5472	1624.1± 46.8	1580	0.0000
	*Cd163*	18.6 ± 1.4	11504	9.8 ± 0.6	12282	N.S.
	*Cd68*	578.4 ± 10.0	3888	957.6± 18.3	2471	0.0000
	Nos2*	3171.8 ± 228.5	870	6261.4± 211.1	510	0.0069
	Arg1*	14.1 ± 1.7	11901	62.2 ± 4.9	9161	0.0000
Primarily	*Cd2*	1174.2 ± 12.6	2211	552.4± 14.5	3636	0.0000
Expressed	*Cd3e*	1994.2 ± 27.7	1359	714.7± 14.6	3033	0.0000
in T-Cells	*Cd8a*	2551.0 ± 33.1	1086	998.0± 12.8	2390	0.0000
	*Cd28*	306.9 ± 5.1	5790	87.8 ± 2.1	8409	0.0000
	*Cd247*	1848.6 ± 18.7	1459	635.4 ± 11.7	3307	0.0000
Primarily	*Cd19*	2206.6 ± 41.6	1250	1126.1 ± 20.5	2181	0.0003
Expressed	Cd22	1362.0 ± 21.3	1943	991.8 ± 19.5	2409	N.S.
in B-Cells	*Cd79b*	4218.4 ± 92.8	658	2214.1 ± 46.3	1221	0.0100
	Cd180**	617.3 ± 12.3	3676	346.3 ± 9.2	4870	0.0008
	*Ly86*	536.7 ± 8.5	4116	379.7 ± 6.7	4620	0.0140
Primarily	*Camp(LL-37)*	1535.7 ± 91.2	1747	1370.5 ± 50.0	1806	N.S.
Expressed	*Elane*	87.2 ± 3.5	8977	207.6 ± 7.7	6259	0.0000
in Neutrophils	*Mpo*	17.1 ± 1.6	11633	11.7 ± 0.8	12003	N.S.
	*Mme*	2.3 ± 0.3	14332	1.3 ± 0.1	15028	N.D.
	*Prtn3*	0.4 ± 0.0	16292	1.3 ± 0.1	14972	N.D.
	*Ctsg*	3.9 ± 0.7	13676	1.6 ± 0.1	14704	N.D.
Expressed	*Cd300a*	968.5 ± 20.4	2589	1575.0 ± 19.3	1626	0.0000
In most	*Cd44*	8363.9 ± 146.3	380	8670.4 ± 84.9	380	N.S.
Leukocytes	*Cd48*	4284.9 ± 48.7	644	2925.8 ± 43.1	957	0.0033
	*Cd84*	252.6 ± 6.5	6362	320.7 ± 6.6	5091	0.0051
	*Cd97*	16286.2 ± 405.0	217	14734.5 ± 209.7	253	N.S.
	*Sell*	18067.8 ± 429.1	194	16497.2 ± 243.4	222	N.S.

*These genes may also be in dendritic cells as well as monocytes but the levels of dendritic cells are much lower in circulating blood than monocytes.

**This gene is also present in plasma cells but the levels of these types of cells in blood would be much lower than B cells in circulating blood.

**Table 3:** Citations for Gene Expression within Leukocyte Cell Types.

Ccr1	chemokine (C-C motif) receptor 1 [49]
Itgax (Cd11c)	integrin, alpha X [50]
Cd14	CD14 molecule [47,50,51]
Cd163	CD163 molecule [51]
Cd68	CD68 molecule [47]
Nos2	nitric oxide synthase 2, inducible [47]
Arg1	arginase 1 [47]
Cd2	CD2 molecule [52]
Cd3e	CD3e molecule [53]
Cd8a	CD8a molecule [54]
Cd28	CD28 molecule [55]
Cd247	CD247 molecule [56]
Cd19	CD19 molecule [53,57,58]
Cd22	CD22 antigen [54]
Cd79b	CD79b molecule [53,57,58]
Cd180	CD180 molecule [53,59]
Ly86	lymphocyte antigen 86 [54]
Camp (LL-37)	cathelicidin antimicrobial peptide [48,60]
Elane	elastase, neutrophil expressed [48,60]
Mpo	myeloperoxidase [48,60]
Mme (Cd10)	membrane metallo-endopeptidase [48,60]
Prtn3	proteinase 3 [48,60]
Ctsg	cathepsin G [48,60]
Cd300a	CD300a molecule [61]
Cd44	CD44 molecule [62]
Cd48	CD48 molecule [63]
Cd84	CD84 molecule [64]
Cd97	CD97 molecule [65]
Sell	selectin L [66]

**Fig 4:**
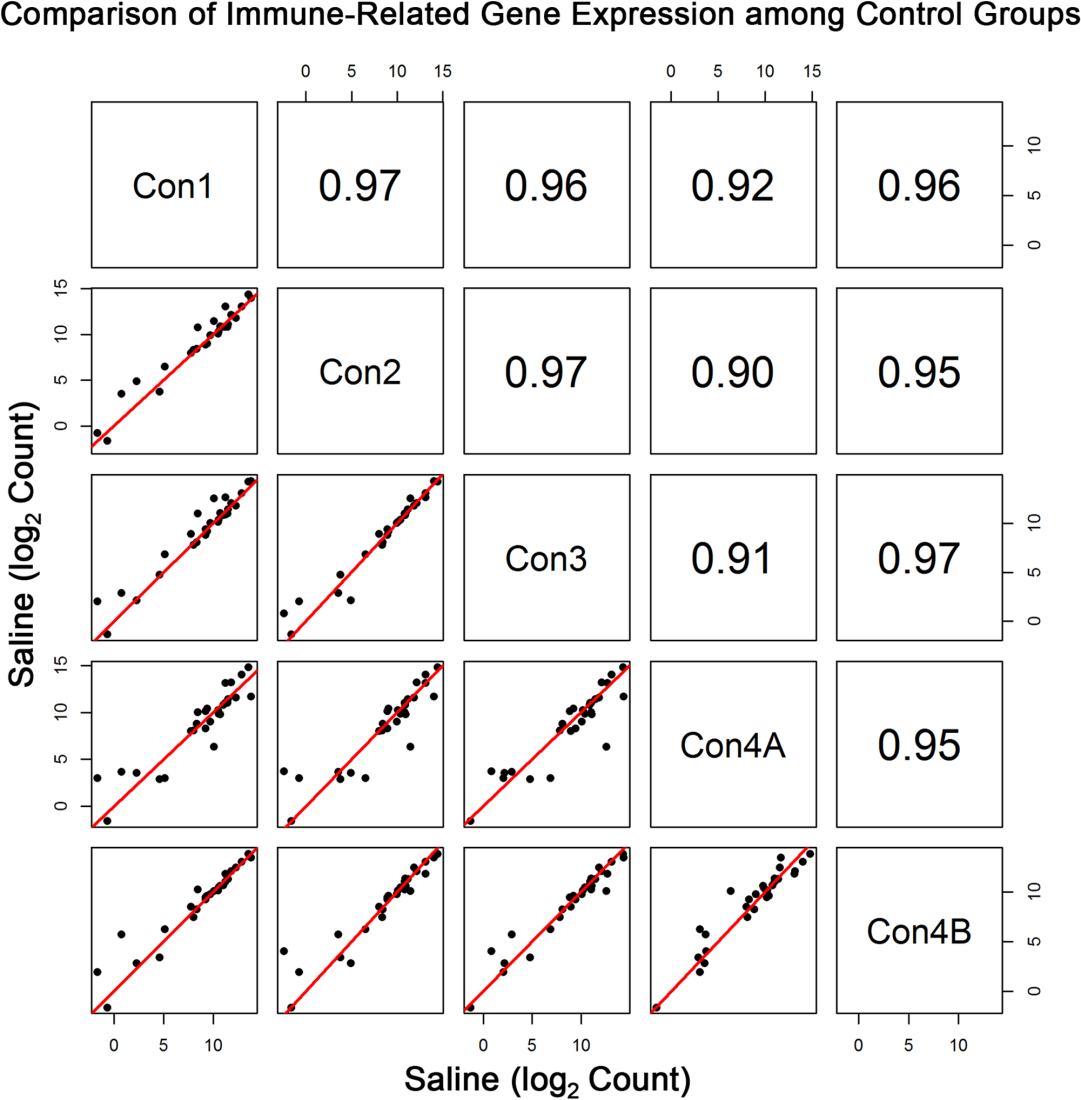
Comparison of Immune-Related Transcripts among the Control Groups. Pearson correlation and scatterplot matrix of log2 normalized expression of the selected immune-related genes in control groups, Con1-Con4B. The red solid line is the identity line.

**Fig 5:**
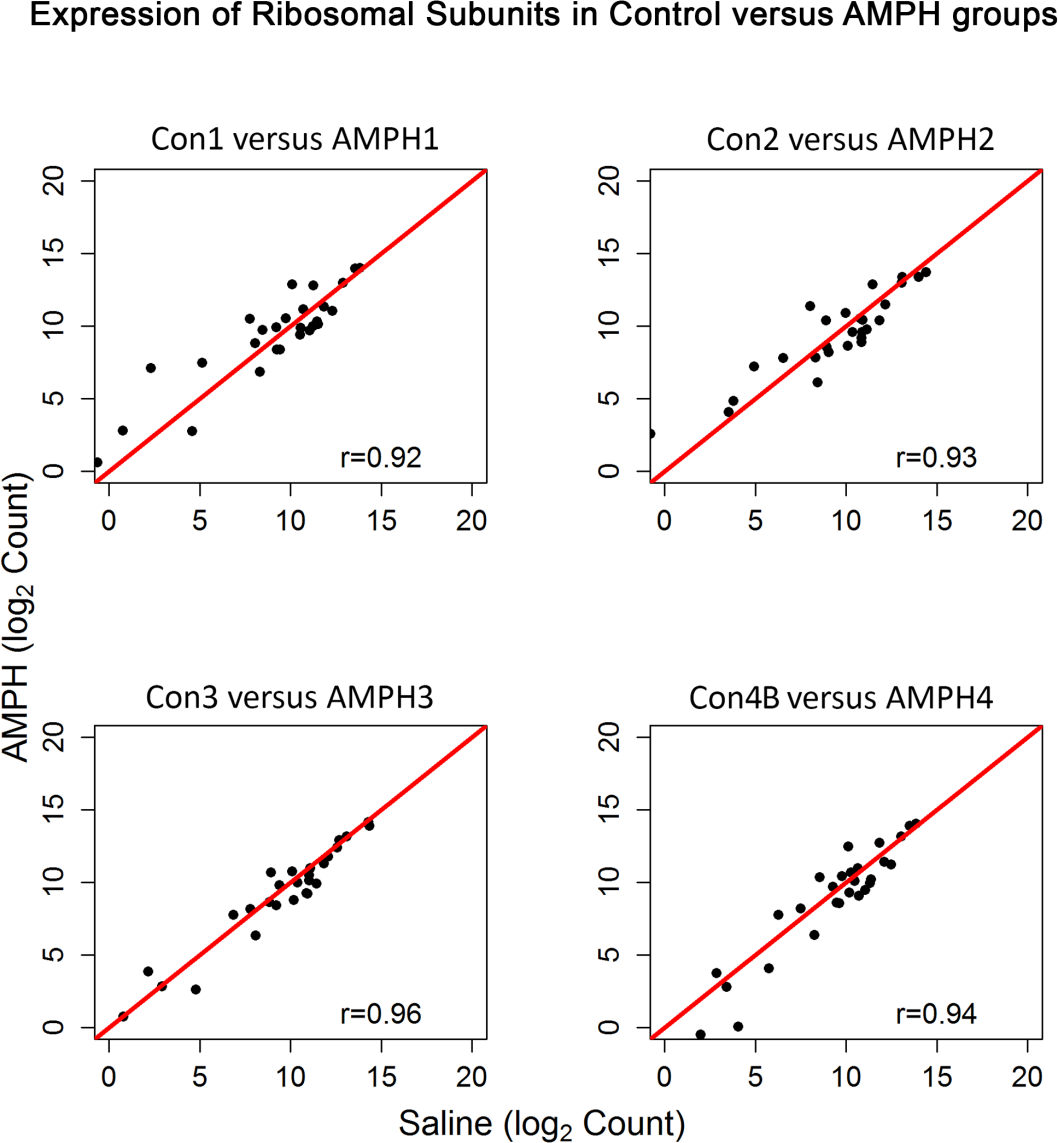
Expression of Immune-Related Genes in the Control versus Amphetamine Groups. Pearson correlation and scatterplot matrix of log2 normalized expression of the selected immune-related genes comparing each control group to its time-matched AMPH group. The red solid line is the identity line.

**Fig 6:**
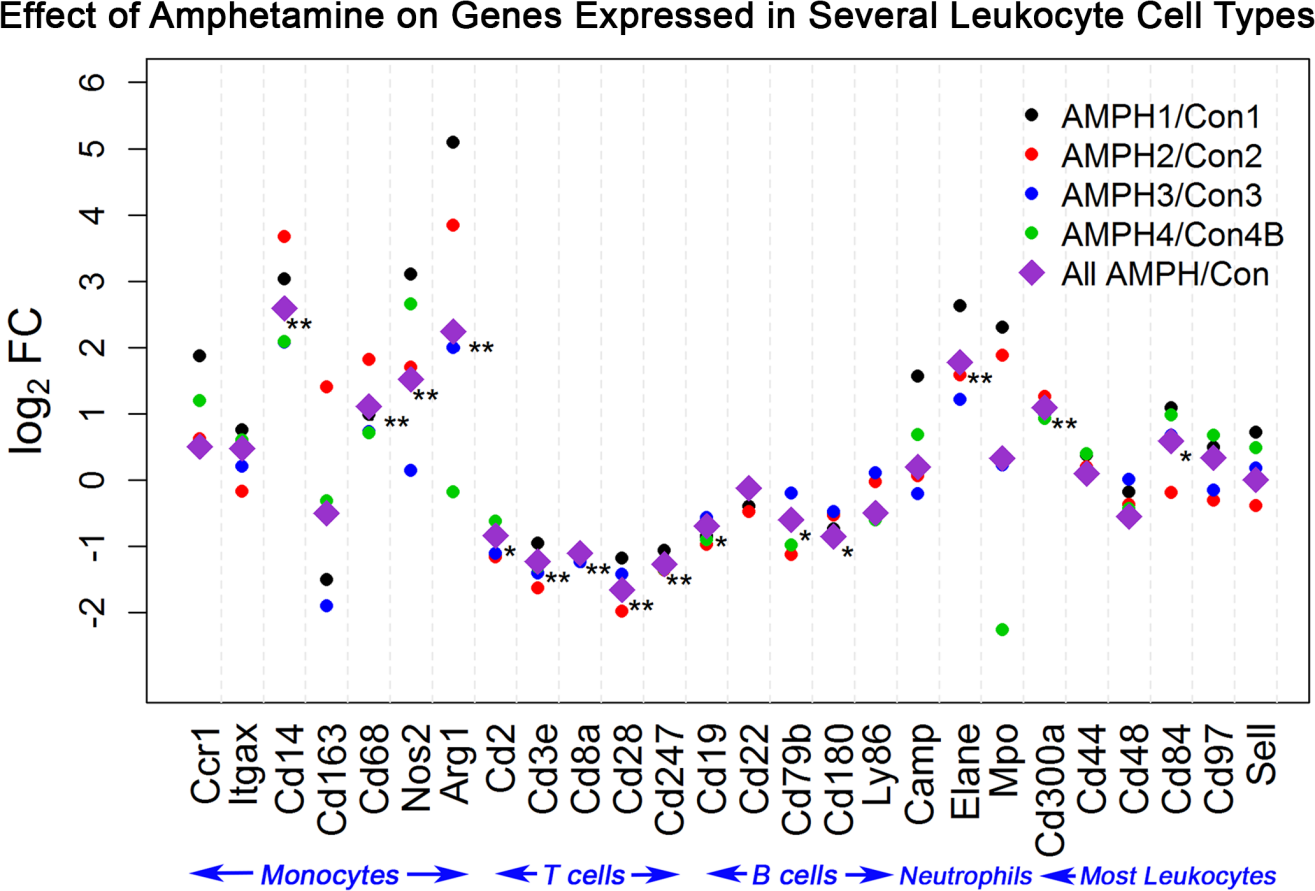
Expression Changes in Genes Primarily Expressed in Monocytes, B-cells or T-cells, and all Leukocytes as a Result of Neurotoxic AMPH Exposure. Scatterplot of log2 fold change (AMPH to control) expression of the selected immune-related genes. For each gene, values of log2FC are presented for each time-matched subgroup comparison using a different colored dot for each subgroup (AMPH1/Con1, black; AMPH2/Con2, red; AMPH3/Con3, blue and AMPH4/Con4B green). The pooled expression of AMPH to control over all subgroups (AMPH/Con) is represented by a purple diamond. Differential expression was assessed for the latter comparison. Log2FC expression for genes with adjusted p-values smaller than 0.05 and 0.58 < |log2FC| < 1 is marked by one asterisk. Likewise, expression for genes with adjusted p-values smaller than 0.05 and |log2FC|≥ 1 is marked with two asterisks.

The expression of these immune-related genes had near-1 correlation (r≥0.97) among the four control groups (Con1-Con3 and Con4B) (Fig 4). However, as expected, the correlation between each control group and its paired time-matched AMPH group was not as strong because of the known effect of the drug on the immune system (Fig 5). Fig 6 presents the log_2_ fold change of AMPH to control expression for the selected immune-related genes, except for the three neutrophil-related genes with very low expression. Relative expression is presented for all four time-matched groups and also for all of the data (denoted by a purple diamond). AMPH has a significant effect on 15 of the 26 genes. Relative to control, all of the genes primarily expressed in T-cells decreased approximately 1.5- to 3.0-fold, while 4 of the 7 monocyte-specific transcripts increased between 2.0 and 6-fold. Expression of 3 of the 5 genes relatively specific for B-cells decreased around 1.5-fold, and genes that are normally expressed in most leukocytes were not significantly different (less 1.5-fold), with only two of the six genes (Cd300a and *Cd84*) reaching significance between AMPH and control.

The levels of the major types of leukocytes in blood for control and AMPH are presented in Table 4. The levels of the lymphocytes (72.3%), neutrophils (23.6%), monocytes (3.5%), eosinophils (0.4%) and basophils (0.2%) were as would be expected based on standard reported levels Monocytes were significantly upregulated by AMPH; the levels of neutrophils were also upregulated by AMPH and marginally significant. Lymphocytes were significantly down-regulated by AMPH.

**Table 4:** Analysis of Leukocyte Cell Types Present in the Whole Blood of Control and Amphetamine Treated Rats.

Cell Type	Control ^b^	AMPH ^a^	Reference for Charles River Control ^b^
Total Leukocytes (WBC)	10.60 ± 1.00	9.93 ± 1.03	10.83 ± 3.84
Lymphocytes (T- and B-cells)	7.65 ± 0.69	5.55 ± 0.57*	6.72 ± 2.53
Neutrophils	2.51 ± 0.49	3.57 ± 0.48*	3.31 ± 1.67
Monocytes	0.37 ± 0.06	0.90 ± 0.14*	0.67 ± 0.33
Eosinophils	0.04 ± 0.01	0.03 ± 0.01	0.13 ± 0.13
Basophils	0.02 ± 0.00	0.06 ± 0.02	0.03 ± 0.04
Erythrocytes (RBC)	7.83 ± 0.34	8.44 ± 0.24	7.6 ± 1.17

^a^ Values for the total and various cell types of leukocytes are measured in thousands (10^3^) per cubic mm of blood and erythrocyte levels are given in millions (10^6^) per cubic mm of blood. Reported values represent mean ± SEM for blood samples collected from 16 control and 16 AMPH animals.

^b^ Reference values for the total and various cell types of leukocytes and RBCs for 56 to 70 day old adult male Charles River Crl:CD(SD) rats were obtained from http://www.criver.com/files/pdfs/rms/cd/rm_rm_d_cd_rat.aspx. The mean ± standard deviation levels are given.

*p < 0.05; Mann-Whitney U test.

## Discussion

The results of our study demonstrate the stability of whole blood RNA-seq transcriptome profiles after globin transcript depletion. Globin depletion was implemented since it has been observed to improve the transcriptome profile in whole blood in previous studies using RNA-seq methods [2]. Any loss of RNA due to globin removal was reasonably consistent since comparisons of transcriptome profiles over a period of two years were highly correlated for both the control and treatment group. The level of agreement is also attributed to consistent expression of each experimental group across time. Our study provides evidence that the consistency and repeatability of RNA-seq of whole blood can be validated by profiling expression of the ribosomal subunits. However, further studies will be necessary to determine whether this approach is more sensitive than methods such as the detection of aberrant G-C profiles [28]. The utility of RNA-seq data from whole blood was further demonstrated by the completeness of the expression profile of immune-related genes as well as the consistent effect that drug treatment had on the expression of these genes, at least in the case of a toxic exposure to AMPH [29].

The 80s ribosome is a group of more than 70 transcripts, of which are all expressed at relatively very high levels [14]. Note that there are 10 transcripts with lower expression; however, the gene for each of these transcripts produced another transcript (splice variant) that measured at much higher levels. As seen in this study, the one anomalous group of RNA-seq data (Con4A) that was less than optimal showed large variability in these transcript levels (Fig 1). This was solely due to a very rare instance of amplification kit variability. Although the observed variability in Con4A expression was somewhat apparent in the entire transcriptome (Fig 2), the deviant variation was readily apparent when profiling the ribosomal subunits. As an example, Pearson correlation values for Con4A were at least 0.90 for the whole genome but were as low as 0.74 when comparing expression levels of the ribosomal transcripts.

An additional benefit of using ribosomal transcripts rather than the entire genome is that the majority of ribosomal transcripts do not change much with AMPH treatment. Only two of the ribosomal transcripts were significantly differentially expressed between control and AMPH (p<0.05 and FC>2). However, it is not yet known how other types of drug/toxicant treatments will affect subunit expression relative to control. Several ribosomal proteins have been used as “housekeeping” genes to normalize RT-PCR expression across individual RNA samples, although they may not be optimal in all tissues [11–14]. As an example, in our study, ribosomal protein L7, Rpl7, was identified as the highest consistently expressed transcript across all 41 samples. Finally, a relatively high expression for at least one transcript of each gene in the ribosomal subunit is almost as important as consistent expression. This is because expression of all of the genes is necessary to form one of the most numerous protein complexes in an organism. Our data showed that at least one transcript for each ribosomal gene ranked in the upper 5^th^ percentile of expression. One could argue that the use of a set of 10 or so genes commonly used to normalize RT-PCR data would provide a better way to determine the potential for significant variation in whole blood RNA-seq. However, such analysis would be limited in some respects because the specific genes in a set of housekeeping genes for RT-PCR are not linked by a common process or protein complex. Thus, unlike the set of ribosomal transcripts, it is much more difficult to determine whether the relative levels of the various genes previously used to normalize RT-PCR are as they should be (in relationship to each other).

In this study, we profiled the expression of immune-related genes (mostly cell determinant genes that are expressed on leukocytes). The stability and consistency of the expression of these immune-related genes are also important as these are the genes in whole blood, along with cytokines and chemokines protein levels in blood, that are used to evaluate how the immune system is responding to a given disease, treatment or toxic insult [30–37]. These genes are major effectors in the chemokine and cytokine signaling system of leukocytes. There have been previous reports on how gene expression, as determined by microarray or RNA-seq, is affected in human blood in anorexic patients or after acute ethanol exposure [38,39]. However, these studies focused primarily on genes not necessary directly related to cytokines, chemokines, or receptors that are exclusively or predominately localized to leukocytes. One very recent RNA-seq study investigated the effects of immunosuppression on the expression of genes related to cytokine/chemokine signaling in mononuclear cells isolated from peripheral blood in patients involved in [40]. A pronounced 10-fold or more down regulation of these genes was observed due to the immunosuppression. Our data clearly indicates that RNA-seq methods are also a sensitive method for detecting changes in gene expression related to cytokine and chemokine signaling resulting from toxic exposures, in this case AMPH.

We observed that the levels of transcripts relatively specific for monocytes, T-cells, B-cells, neutrophils or present in all leukocytes were roughly as expected in the circulating blood of rat. Transcript levels relatively specific for monocytes, the least abundant cell type (3%), were readily detectable in the control and amphetamine groups, and some such as *Ccr1* had very high expression. All but one of the genes specific for neutrophils, which are 8 to 15% of the all WBCs in rat, were at low levels. This would be expected due to the nature of mature neutrophils present in circulating blood. However, some genes, mostly those also present in other types of WBCs, can be induced [19]. Therefore, some of the expression that we observed in blood of interleukin 1b (*Il1b*) and neutrophil cytosolic factor 1 (*Ncf1*), which were at very high levels (data not shown), could have been due to expression in neutrophils as well as monocytes. Thus, the overall profile of immune expression was well represented.

There was a significant difference between the control and amphetamine groups for several of the selected immune-related genes. Specifically, 10 of the 26 immune-related genes had 2-fold or greater change in expression and 5 additional genes demonstrated at least 1.5-fold change in expression. Two genes, *Cd14* and *Arg1*, increased expression more than 4-fold as a result of amphetamine exposure. Importantly, the response (change in transcript levels) of all these genes, except *Cd163*, was very consistent across all four treatment groups. In general, many of the changes observed in these immune related genes might be expected to be due to AMPH exposure, including the increase in β-adrenergic stimulation produced by AMPH releasing norepinephrine [41–44]. Finding treatment-induced changes in immune-related transcripts is of the utmost importance since previous, and likely subsequent, animal studies involving gene expression in blood or serum have focused on changes related to immune system function.

Our data on cell counts of monocytes and lymphocytes (T-cells and B-cells) in circulating blood indicate that the transcript expression changes may be partially explained by increased or decreased numbers of specific types of leukocytes present in circulating blood after AMPH. The monocyte count increased slightly over two fold while the lymphocyte levels decreased approximately 30%. In regards to the monocytes, the increase in their numbers could partially explain the increase in expression of genes specifically found in them. Nonetheless, the changes in numbers/counts cannot explain all the gene expression changes seen. The expression increases in monocyte-specific genes range from a less than 1.5-fold increase (*Ccr1* and *Itgax*) to almost a 6-fold increase for *Cd14*, which would strongly argue that there is likely a relative change in the relative expression of the genes present within the circulating monocytes. Likewise, the 30% decreases in the number of circulating T-cells and B-cells may not explain the much greater fold change decreases, particularly in T-cell-related genes. Overall, the increases in the circulating blood expression profile and monocyte and neutrophil cells numbers indicate an activation of the innate immune system by AMPH [45–48].

The stability of transcript expression in four control groups of animals (Sprague-Dawley rats in this study) over a two-year timeframe was strong. However, more importantly, the changes in gene expression of the animals given a neurotoxic dosage of AMPH relative to the controls were very consistent across all four time points. Thus, the work presented here demonstrates that under these conditions control and treatment-specific animals can be pooled for analysis. The increase in sample size will subsequently increase power to detect differentially expressed transcripts. The ability to show reproducibility of treatment effect on the transcriptome over time lends to the validation of the expression changes that are reported.
